# Uptake and outcome of manuscripts in Nature journals by review model and author characteristics

**DOI:** 10.1186/s41073-018-0049-z

**Published:** 2018-08-17

**Authors:** Barbara McGillivray, Elisa De Ranieri

**Affiliations:** 1 The Alan Turing Institute, London, England; 2 Theoretical and Applied Linguistics, Faculty of Modern and Medieval Languages, University of Cambridge, Cambridge, UK; 3 Springer Nature, 4 Crinan Street, London, UK

**Keywords:** Double-blind peer review, Peer review bias, Gender bias, Acceptance rate, Nature journals, Implicit bias

## Abstract

**Background:**

Double-blind peer review has been proposed as a possible solution to avoid implicit referee bias in academic publishing. The aims of this study are to analyse the demographics of corresponding authors choosing double-blind peer review and to identify differences in the editorial outcome of manuscripts depending on their review model.

**Methods:**

Data includes 128,454 manuscripts received between March 2015 and February 2017 by 25 Nature-branded journals. We investigated the uptake of double-blind review in relation to journal tier, as well as gender, country, and institutional prestige of the corresponding author. We then studied the manuscripts’ editorial outcome in relation to review model and author’s characteristics. The gender (male, female, or NA) of the corresponding authors was determined from their first name using a third-party service (Gender API). The prestige of the corresponding author’s institutions was measured from the data of the Global Research Identifier Database (GRID) by dividing institutions in three prestige groups with reference to the 2016 Times Higher Education (THE) ranking. We employed descriptive statistics for data exploration, and we tested our hypotheses using Pearson’s chi-square and binomial tests. We also performed logistic regression modelling with author update, out-to-review, and acceptance as response, and journal tier, author gender, author country, and institution as predictors.

**Results:**

Author uptake for double-blind submissions was 12% (12,631 out of 106,373). We found a small but significant association between journal tier and review type (*p* value < 0.001, Cramer’s *V* = 0.054, df = 2). We had gender information for 50,533 corresponding authors and found no statistically significant difference in the distribution of peer review model between males and females (*p* value = 0.6179). We had 58,920 records with normalised institutions and a THE rank, and we found that corresponding authors from the less prestigious institutions are more likely to choose double-blind review (*p* value < 0.001, df = 2, Cramer’s *V* = 0.106). In the ten countries with the highest number of submissions, we found a large significant association between country and review type (*p* value < 0.001, df = 10, Cramer’s *V* = 0.189). The outcome both at first decision and post review is significantly more negative (i.e. a higher likelihood for rejection) for double-blind than single-blind papers (*p* value < 0.001, df = 1, Cramer’s *V* = 0.112 for first decision; *p* value < 0.001; df = 1, Cramer’s *V* = 0.082 for post-review decision).

**Conclusions:**

The proportion of authors that choose double-blind review is higher when they submit to more prestigious journals, they are affiliated with less prestigious institutions, or they are from specific countries; the double-blind option is also linked to less successful editorial outcomes.

## Background

Double-blind peer review (DBPR) has been proposed as a means to avoid implicit bias from peer reviewers against characteristics of authors such as gender, country of origin, or institution. Whereas in the more conventional single-blind peer review (SBPR) model, the reviewers have knowledge of the authors’ identity and affiliations [1]; under DBPR, the identity and affiliations of the authors are hidden from the reviewers and vice versa. In spite of the presence of explicit instructions to authors, this type of review model has sometimes been shown to fail to hide authors’ identity. For example, a report showed that 34% of 880 manuscripts submitted to two radiology journals contained information that would either potentially or definitely reveal the identities of the authors or their institution [2]. Another report found that the authors of submissions to the American Journal of Public Health were in fact recognizable in around half of the cases [3].

Over the past years, several studies have analysed the efficacy of DBPR in eradicating implicit bias in specific scientific disciplines. In a systematic review and meta-analysis of biomedical journals investigating the interventions aimed at improving the quality of peer review in these publications, the authors reported that DBPR “did not affect the quality of the peer review report or rejection rate” [4]. Similar results were reported for the journal *Plastic and Reconstructive Surgery* [5]. Some research has not found conclusive results [6, 7], demonstrating the need for further large-scale systematic analyses spanning over journals across the disciplinary spectrum. Regarding gender bias, a study showed that blinding interviewees in orchestra interviews led to more females being hired [8]. In the context of scientific literature, an analysis of 2680 manuscripts from seven journals found no overall difference in the acceptance rates of papers according to gender, while at the same time reporting a strong effect of number of authors and country of affiliation on manuscripts’ acceptance rates [9]. A study of the distribution of gender among reviewers and editors of the Frontiers journals showed an underrepresentation of women in the process, as well as a same-gender preference (homophily) [10]. An analysis of the journal *Behavioral Ecology*, which switched to DBPR in 2001, found a significant interaction between gender and time, reflecting the higher number of female authors after 2001, but no significant interaction between gender and review type [11]. A study analysing 940 papers submitted to an international conference on economics held in Sweden in 2008 found no significant difference between the grades of female- and male-authored papers by review type [12]. On the other hand, an analysis of the Evolution of Language (EvoLang 11) conference papers found that female authors received higher rankings under DBPR [13]. Among the studies dealing with institutional bias, an analysis of abstracts submitted to the American Heart Association’s annual Scientific Sessions research meeting from 2000 to 2004 found some evidence of bias favouring authors from English-speaking countries and prestigious institutions [14].

Regarding institutional bias, a report of a controlled experiment found that SBPR reviewers are more likely than DBPR reviewers to accept manuscripts from famous authors and high-ranked institutions [15], while another report found that authors at top-ranked universities are unaffected by different reviewing methods [16].

The study reported on here is the first one that focusses on Nature-branded journals, with the overall aim to investigate whether there is any implicit bias in peer review in these journals and ultimately understand whether DBPR is an effective measure in removing referee bias and improving the peer review of scientific literature. We focus on the Nature journals as that portfolio covers a wide range of disciplines in the natural sciences and biomedical research, and thus, it gives us an opportunity to identify trends beyond discipline-specific patterns. In addition, the high prestige of these journals might accentuate an implicit referee bias and therefore makes such journals a good starting point for such an analysis.

Nature-branded journals publishing primary research introduced DBPR as an optional service in March 2015 in response to authors’ requests [17]. At the point of first submission, authors have to indicate whether they wish to have their manuscript considered under SBPR or DBPR, and this choice is maintained if the manuscript is declined by one journal and transferred to another. If authors choose DBPR, their details (names and affiliations) are removed from the manuscript files, and it is the authors’ responsibility to ensure their own anonymity throughout the text and beyond (e.g. authors opting for DBPR should not post on preprint archives). Editors are always aware of the identity of the authors.

We note here that, in recent years, trends in scholarly publishing have emerged that strongly propose transparent, or open, peer review as a model that could potentially improve the quality and robustness of the peer review process [18]. There is not yet sufficient data to conclude which form of peer review—transparent or double-blind—is the most conducive to rigorous and unbiased science reporting. Moreover, the two models do not have to be exclusive; one could think of a DBPR stage followed by full public disclosure of reviewers’ and editor’s identities and reports. The present study focusses on the effects of this publisher intervention in the 2 years following implementation and can guide others when evaluating the consequences of introducing DBPR to their journals.

In this study, we sought to understand the demographics of authors choosing DBPR in Nature-branded journals and to identify any differences in success outcomes for manuscripts undergoing different review models depending on the gender and the affiliation of the corresponding author.

## Methods

The study was designed to analyse the manuscripts submitted to Nature-branded journals publishing primary research between March 2015 (when the Nature-branded primary research journals introduced DBPR as an opt-in service) and February 2017.

For each manuscript, we used Springer Nature’s internal manuscript tracking system to extract name, institutional affiliation, and country of the corresponding author; journal title; the manuscript’s review type (single-blind or double-blind); the editor’s final decision on the manuscript (accept, reject, or revise); and the DOI. Here, we define the corresponding author as the author who is responsible for managing the submission process on the manuscript tracking system and for all correspondence with the editorial office prior to publication. We have used this definition because it is in line with that used in the guide to authors for Nature (https://www.nature.com/nature/for-authors/initial-submission). Please note that this definition is different from that of the corresponding author(s) as stated on published articles and who are the author(s) responsible for correspondence with readers.

The dataset consisted of 133,465 unique records, with 63,552 different corresponding authors and 209,057 different institution names. In order to reduce the variability in the institutional affiliations, we normalised the institution names and countries via a Python script that queried the API of the Global Resource Identified Database (GRID [19]). We only retained a normalised institution name and country when the query to the GRID API returned a result with a high confidence, and the flag “manual review” was set to false, meaning that no manual review was needed. This process left 13,542 manuscripts without a normalised name; for the rest of the manuscripts, normalised institution names and countries were found, which resulted in 5029 unique institution names.

In order to assign a measure of institutional prestige to each manuscript, we used the 2016/2017 Times Higher Education rankings (THE [20]) and normalised the institution names using the GRID API. We then mapped the normalised institution names from our dataset to the normalised institution names of the THE rankings via a Python script. Finally, we associated each author with a gender label (male/female) by using the Gender API service [21].

The final dataset was further processed and then analysed statistically using the statistical programming language R, version 3.4.0. In the processing step, we excluded 5011 (3.8%) records which had an empty value in the column recording the review type due to technical issues in the submissions system for Nature Communications. These records are excluded from the analysis, resulting in a dataset of 128,454 records, of which 20,406 (16%) were submitted to Nature, 65,234 (51%) to the 23 sister journals, and 42,814 (33%) to Nature Communications.

The dataset contains both direct submissions and transfers, i.e. manuscripts originally submitted to a journal and subsequently transferred to another journal which was deemed a better fit by the editor. In the case of transfers, the author cannot change the review type compared to the original submission, and therefore, we excluded the 22,081 (17%) transferred manuscripts from the analysis of author uptake. We however included transfers in all other analyses because we considered the analysed items as combinations of three attributes: paper, corresponding author, and journal to which the paper was submitted. Table 1 displays the number and proportion of transfers by journal group.

**Table 1:** Number of transferred manuscripts and direct submissions received by each journal group

Journal group	Transfers	Direct submissions
Nature	57 (0.3%)	20,349 (99.7%)
Sister journals	9446 (14.5%)	55,788 (85.5%)
Nature Communications	12,578 (29.4%)	30,236 (70.6%)

We inspected the gender assigned via the Gender API, which assigns an accuracy score between 0 and 100 to each record. After manually checking a sample of gender assignments and their scores, we kept the gender returned by Gender API where the accuracy was at least 80 and assigned a value “NA” otherwise. This resulted in 17,379 (14%) instances of manuscripts whose corresponding author was female, 83,830 (65%) manuscripts with male corresponding author, and 27,245 (21%) manuscripts with gender NA. In the following analysis, we will refer to the data where the gender field is not NA as the Gender Dataset.

Concerning the institutions, we defined four categories according to their THE ranks and used these as a proxy for prestige: category 1 includes institutions with THE rank between 1 and 10 (corresponding to 7167 manuscripts, 6% of all manuscripts), category 2 is for THE ranks between 11 and 100 (25,345 manuscripts, 20% of all manuscripts), category 3 for THE ranks above 100 (38,772 manuscripts, 30% of all manuscripts), and category 4 for non-ranked institutions (57,170 manuscripts, or 45% of all manuscripts). This choice of categories is arbitrary, e.g. we could have chosen a different distribution of institutions among the four categories, and will likely have an impact on the uptake of DBPR across the institutional prestige spectrum. However, we find that a logarithmic-based categorization of this sort would be more representative than a linear-based one. In the following analysis, we will refer to the data for groups 1, 2, and 3 as the Institution Dataset.

We employed hypothesis testing techniques to test various hypotheses against the data. In order to test whether two variables were independent, we used Pearson’s chi-square test of independence and referred to the classification in [21] to define the strength of association. In order to test whether the proportions in different groups were the same, we used the test of equal proportions in R (command “prop.test”). We used a significance threshold of 0.05.

We also conducted regression analyses on the data, to measure the effect of different variables such as gender and institution group on three outcomes: author uptake, out-to-review, and acceptance. We fitted logistic regression models and report details on their goodness of fit.

## Results

We analysed the dataset of 128,454 records with a non-empty review type to answer the following questions:

1. What are the demographics of authors that choose double-blind peer review?
2. Which proportions of papers are sent out to review under SBPR and DBPR? Are there differences related to gender or institution within the same review model?
3. Which proportions of papers are accepted for publication under SBPR and DBPR? Are there differences related to gender or institution within the same review model?

To place the results below within the right context, we point out that this study suffered from a key limitation, namely that we did not have an independent measure of quality for the manuscript or a controlled experiment in which the same manuscript is reviewed under both peer review models. As a consequence, we are unable to distinguish bias towards author characteristics or the review model from any quality effect, and thus, we cannot draw definitive conclusions on the efficacy of DBPR in addressing bias. We discuss the limitations of the study in more detail in the “Discussion” section.

### Analysis of peer review model uptake

We first analysed the demographics of corresponding authors that choose DBPR by journal group, gender, country, and institution group. When analysing data for the entire portfolio, we only included direct submissions (106,373) and we excluded manuscripts that were rejected by one journal and then transferred to another. This is because authors cannot modify their choice of review model at the transfer stage, and thus transfers cannot contribute to the uptake analysis. The overall uptake of DBPR is 12%, corresponding to 12,631 manuscripts, while for 93,742 manuscripts, the authors chose the single-blind option.

When analysing uptake data by journal tier, we have included both direct submissions and transfers incoming to each journal group, for a total of 128,457 manuscripts that were submitted to one of the 25 Nature-branded journals. We investigated any potential differences in uptake depending on the journal tier. We divided the journals in three tiers: (i) the flagship interdisciplinary journal (Nature), (ii) the discipline-specific sister journals (Nature Astronomy, Nature Biomedical Engineering, Nature Biotechnology, Nature Cell Biology, Nature Chemical Biology, Nature Chemistry, Nature Climate Change, Nature Ecology & Evolution, Nature Energy, Nature Genetics, Nature Geoscience, Nature Human Behaviour, Nature Immunology, Nature Materials, Nature Medicine, Nature Methods, Nature Microbiology, Nature Nanotechnology, Nature Neuroscience, Nature Photonics, Nature Physics, Nature Plants, Nature Structural & Molecular Biology), and (iii) the open-access interdisciplinary title (Nature Communications).

Table 2 displays the uptake by journal group and shows that the review model distribution changes as a function of the journal tier, with the proportion of double-blind papers decreasing for tiers with comparatively higher perceived prestige. We found a small but significant association between journal tier and review type. The results of a Pearson’s chi-square test of independence are as follows: *χ*^2^ = 378.17, degrees of freedom = 2, *p* value < 0.001; Cramer’s *V* = 0.054 and show that authors submitting to more prestigious journals tend to have a slight preference for DBPR compared to SBPR. This might indicate that authors are more likely to choose DBPR when the stakes are higher in an attempt to increase their success chances by removing any implicit bias from the referees.

**Table 2:** Uptake of peer review models by journal tier

Journal group	DBPR	SBPR
Nature	2782 (14%)	17,624 (86%)
Sister journals	8053 (12%)	57,181 (88%)
Nature Communications	3900 (9%)	38,914 (91%)

We then analysed the uptake by gender for the entire portfolio, as we were interested in finding any gender-related patterns. Table 3 shows the distribution of DBPR and SBPR in the three gender categories.

**Table 3:** Uptake of peer review models by gender of the corresponding author

Gender	DBPR	SBPR
Female	1506 (10%)	12,943 (90%)
Male	7271 (11%)	61,536 (89%)
NA	3854 (17%)	19,263 (83%)

We only considered 83,256 (out of the 106,373) manuscripts for which the gender assigned to the corresponding author’s name by Gender API had a confidence score of at least 80 and the gender was either male or female (the Gender Dataset, excluding transfers). We did not find a significant association between gender and review type (Pearson’s chi-square test results: *χ*^2^ = 0.24883, df = 1, *p* value = 0.6179).

In order to see if institutional prestige played a role in the choice of review type by authors, we analysed the uptake by institution group for the entire portfolio. For this analysis, we used a subset of the 106,373 manuscripts consisting of 58,920 records with non-empty normalised institutions for which a THE rank was available (the Institution Dataset, excluding transfers) (Table 4).

**Table 4:** Uptake of peer review models by institution group

Institution group	DBPR	SBPR
	Actual	Actual
1	240 (4%)	5818 (96%)
2	1663 (8%)	19,295 (92%)
3	4174 (13%)	27,730 (87%)

We investigated the relationship between review type and institutional prestige (as measured by the institution groups) by testing the null hypothesis that the review type is independent from prestige. A Pearson’s chi-square test found a significant, but small association between institution group and review type (*χ*^2^ = 656.95, df = 2, *p* value < 0.001, Cramer’s *V* = 0.106). We can conclude that authors from the least prestigious institutions are more likely to choose DBPR compared to authors from the most prestigious institutions and authors from the mid-range institutions.

Finally, we investigated the uptake of the peer review models by country of the corresponding author for the entire portfolio, using data on all of the 106,373 manuscripts. We found that 10 countries contributed to 80% of all submissions, and thus, we grouped all other countries under the category “Others”. Results on the uptake are shown in Table 5.

**Table 5:** Uptake of peer review model by country

Country	DBPR	SBPR
Australia	274 (10%)	2366 (90%)
Canada	259 (9%)	2581 (91%)
China	3626 (22%)	13,148 (78%)
France	278 (8%)	3334 (92%)
Germany	350 (5%)	6079 (95%)
India	711 (32%)	1483 (68%)
Japan	933 (15%)	5248 (85%)
South Korea	643 (12%)	3089 (88%)
UK	509 (7%)	6656 (93%)
USA	2298 (7%)	30,184 (93%)
Others	2750 (12%)	19,574 (88%)

**Fig. 1:**
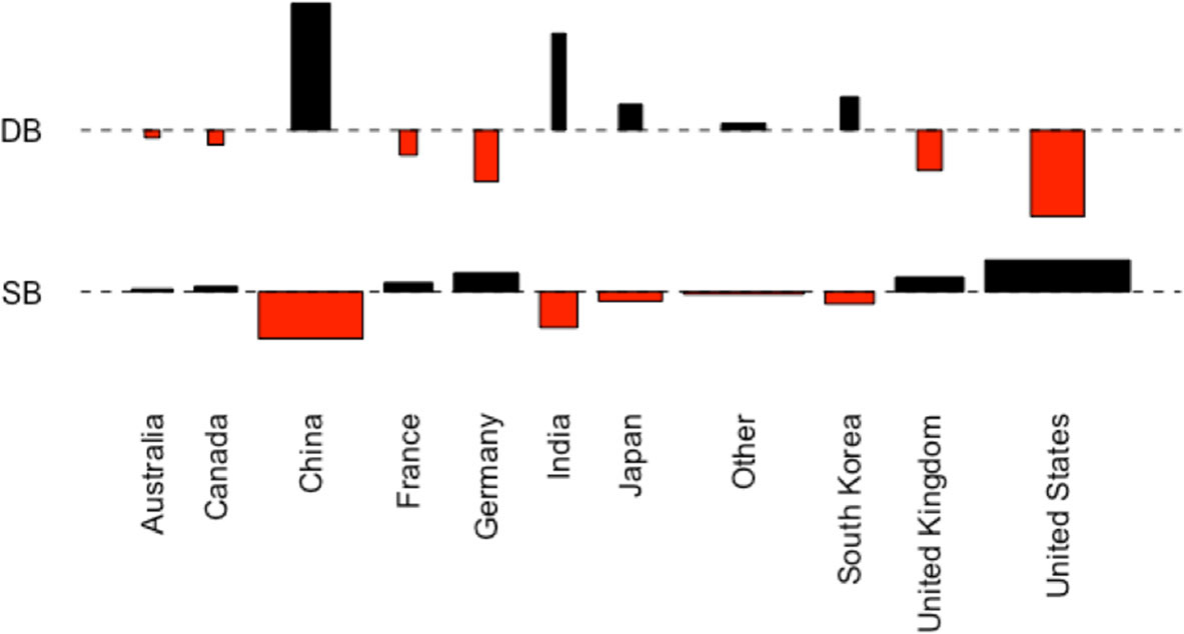
Cohen-Friendly association plot for Table 5

Using Pearson’s chi-square test of independence, we found a significant and large association between country category and review type (*χ*^2^ = 3784.5, df = 10, *p* value < 0.001; Cramer’s *V* = 0.189). Figure 1 shows a Cohen-Friendly association plot indicating deviations from independence of rows (countries) and columns (peer review model) in Table 5. The area of each rectangle is proportional to the difference between observed and expected frequencies, where the dotted lines refer to expected frequencies. The height of the rectangles is related to the significance and the width to the amount of data that support the result. China and the USA stand out for their strong preference for DBPR and SBPR, respectively.

In order to see whether author uptake could be accurately predicted based on author and journal characteristics, we attempted to fit logistic regression models to the data. We aimed at modelling uptake (baseline SB) based on the following variables (and all their subsets): corresponding author’s gender, the group of their institution (1, 2, 3, or 4), the category of their country (Australia, Canada, China, France, Germany, India, Japan, South Korea, the UK, the USA, and Others), and the journal tier (Nature, Nature sister journals, and Nature Communications). However, we did not find a combination of predictors that led to a model with a good fit to the data. The full model has a pseudo *R*^2^ of 0.06, which means that the model only represents a 6% improvement over simply guessing the most frequent outcome, or in other words, the model is not powerful enough to predict the uptake of DB with high reliability. The binned plot of the model’s residuals against the expected values also shows a poor fit. The area under the receiving operating characteristic (ROC) curve is as low as 0.33, indicating that other explanatory variables should be included. As a matter of fact, the model’s accuracy (as tested on a random sample of 20% of the data chosen as test set) is 0.88, and the model always predicts author choices for SB, which is the majority class. Similar results are achieved if simpler logistic regression models are considered, such as review type modelled on journal tier and institution and review type modelled on journal tier only. The results of a likelihood ratio showed that the more complex model is better than the simpler ones, and its pseudo *R*^2^ is the highest (though very low). We employed a Wald test to evaluate the statistical significance of each coefficient in the model by testing the hypothesis that the coefficient of an independent variable in the model is significantly different from zero. The result was a *p* value below 0.05, which shows that removing any of the predictors would harm the fit of the best model. We also attempted to fit a generalized linear mixed effects model with a random effect for the country category, as we can assume that the data is sampled by country and observations from the same country share characteristics and are not independent. However, we did not achieve a good fit, as per the binned plot of residuals against expected values, and the C-index (used to assess the discriminatory ability of standard logistic models) is 0.68, so well below the threshold of 0.8 for good fit.

### Analysis at the out-to-review stage

Once a paper is submitted, the journal editors proceed with their assessment of the work and decide whether each manuscript is sent out for review (OTR) to external reviewers. This decision is taken solely by the editors, who are aware of the chosen peer review model as well as all author information. We investigated the proportion of OTR papers (OTR rate) under both peer review models to see if there were any differences related to gender or institution. For this analysis, we included direct submissions as well as transferred manuscripts, because the editorial criteria vary by journal and a manuscript rejected by one journal and transferred to another may then be sent out to review. Thus, our unit of analysis is identified by three elements: the manuscript, the corresponding author, and the journal.

Table 6 shows the counts and proportions of manuscripts that were sent out for review or rejected by the editors as a function of peer review model.

**Table 6:** Outcome of the first editorial decision (OTR rate) for papers submitted under the two peer review models

Outcome	DBPR		SBPR	
Rejected outright	13,493 (92%)		87,734 (77%)	
	Nature	2634	Nature	13,499
	Nature Communications	3328	Nature Communications	27,728
	Sister journals	7531	Sister journals	46,507
Out to review	1242 (8%)		25,985 (23%)	
	Nature	148	Nature	4125
	Nature Communications	572	Nature Communications	11,186
	Sister journals	522	Sister journals	10,674

We found that a smaller proportion of DBPR papers are sent to review compared with SBPR papers and that there is a very small but significant association between review type and outcome of the first editorial decision (results of a chi-square test: *χ*^2^ = 1623.3, df = 1, *p* value < 0.001; Cramer’s *V* = 0.112).

We also analysed the OTR rates by gender of the corresponding author, regardless of review type. Here, we included data on direct submissions and transfers (101,209 submissions). We excluded data where the gender was not assigned to either male or female. Table 7 shows the results; for the sake of completeness, Table 7 includes the number and percentages of rejected vs. out-to-review manuscripts for which the gender of the corresponding author was NA.

**Table 7:** Outcome of the first editorial decision (OTR rate) as a function of corresponding author’s gender, regardless of peer review model

Outcome	Female corresponding authors	Male corresponding authors	NA value for gender of corresponding authors
Rejected outright	13,493 (77.6%)	65,046 (77.6%)	22,688 (83.3%)
Out to review	3886 (22.4%)	18,784 (22.4%)	4557 (16.7%)

We did not find a significant association between OTR and gender (Pearson’s chi-square test results: *χ*^2^ = 0.015641, df = 1, *p* value = 0.9005). Our main question concerns a possible gender bias; therefore, we investigated the relation between OTR rates, review model, and gender, still including both direct submissions and transfers (Table 8). For the sake of completeness, Table 8 includes the number and percentages of rejected vs. out-to-review manuscripts for which the gender of the corresponding author was male, female, or NA.

**Table 8:** Outcome of the first editorial decision (OTR rate) as a function of corresponding author’s gender and peer review model

Outcome	Female corresponding authors		Male corresponding authors		NA value for gender of corresponding authors	
	DBPR	SBPR	DBPR	SBPR	DBPR	SBPR
Rejected outright	1549 (89.6%)	11,944 (76.3%)	7835 (91.7%)	57,211 (76.0%)	4109 (92.2%)	18,579 (81.5%)
Out to review	180 (10.4%)	3706 (23.7%)	713 (8.3%)	18,071 (24.0%)	349 (7.8%)	4208 (18.5%)

From inspection of Table 8, it would seem that SBPR manuscripts by female corresponding authors are more likely to be rejected at the first editorial decision stage than those by male corresponding authors and that DBPR manuscripts by male corresponding authors are less likely to be sent to review than those by female corresponding authors. We decided to exclude the NA entries for gender and tested the null hypothesis that the two populations (manuscripts by male corresponding authors and manuscripts by female corresponding authors) have the same OTR rate within each of the two review models. For this, we used a test for equality of proportions with continuity correction. For DBPR papers, we found a statistically significant difference in the OTR rate by gender (*χ*^2^ = 7.5042, df = 1, *p* value = 0.006155); for SBPR papers, we did not find a statistically significant difference in the OTR rate by gender (*χ*^2^= 0.72863, df = 1, *p* value = 0.3933). Therefore, in the DBPR case, we can conclude that there is a significant difference between the OTR rate of papers by male corresponding authors and the OTR rate of papers by female corresponding authors. In the SBPR case, we cannot reject the null hypothesis.

Next, we focussed on a potential institutional bias and looked at the relationship between OTR rate and institutional prestige as measured by the groups defined based on THE ranking explained above (excluding the fourth group, for which no THE ranking was available), regardless of review type (Table 9).

**Table 9:** Outcome of the first editorial decision (OTR rate) as a function of the group of the corresponding author’s institution, regardless of peer review model

Outcome	Institution group 1	Institution group 2	Institution group 3
Rejected outright	4541 (63%)	18,949 (75%)	32,046 (83%)
Out to review	2626 (37%)	6396 (25%)	6726 (17%)

Papers from more prestigious institutions are more likely to be sent to review than papers from less prestigious institutions, regardless of review type. This is a statistically significant result, with a small effect size; the results of Pearson’s chi-square test of independence are as follows: *χ*^2^ = 1533.9, df = 2, *p* value < 0.001, Cramer’s *V* = 0.147. This may be due to the higher quality of the papers from more prestigious institutions or to an editor bias towards institutional prestige, or both.

Next, we investigated the relation between OTR rates, review model, and institution group (Table 10) to detect any bias.

**Table 10:** Outcome of the first editorial decision (OTR rate) as a function of the group of the corresponding author’s institution and the peer review model

Outcome	Institution group 1		Institution group 2		Institution group 3	
	DBPR	SBPR	DBPR	SBPR	DBPR	SBPR
Rejected outright	241 (85.8%)	4300 (62.4%)	1696 (86.8%)	17,253 (73.8%)	4487 (91.8%)	27,559 (81.3%)
Out to review	40 (14.2%)	2586 (37.6%)	259 (13.2%)	6137 (26.2%)	399 (8.2%)	6327 (18.7%)

We observed a trend in which the OTR rate for both DBPR and SBPR papers decreases as the prestige of the institution groups decreases, and we tested for the significance of this. A test for equality of proportions for groups 1 and 2 for DBPR papers showed a non-significant result (*χ*^2^ = 0.13012, df = 1, *p* value = 0.7183), and the same test on group 2 and group 3 for DBPR papers showed a significant result (*χ*^2^ = 40.898, df = 1, *p* value < 0.001). A test for equality of proportions for groups 1 and 2 for SBPR papers returned a significant difference (*χ*^2^ = 331.62, df = 1, *p* value < 0.001); the same test for group 2 and group 3 for SBPR papers also returned a significant difference (*χ*^2^ = 464.86, df = 1, *p* value < 0.001).

In order to see whether the OTR outcome could be accurately predicted based on author and journal characteristics, we attempted to fit logistic regression models to the data. We aimed at modelling OTR decisions based on the following variables (and all their subsets): review type (SB/DB), corresponding author’s gender, the group of their institution (1, 2, 3, or 4), the category of their country (Australia, Canada, China, France, Germany, India, Japan, South Korea, the UK, the USA, and Others), and the journal tier (Nature, Nature sister journals, and Nature Communications). Similar to the uptake case, the models do not have a good fit to the data. The full model has a pseudo *R*^2^ of 0.05, and the binned plot of the model’s residuals against the expected values also shows a poor fit. The area under the receiving operating characteristic (ROC) curve is 0.65.

### Analysis of outcome post-review

Finally, we investigated the outcome of post-review decisions as a function of peer review model and characteristics of the corresponding author. We studied whether papers were accepted or rejected following peer review, and we included transfers because the editorial decisions as different journals follow different criteria. We excluded papers for which the post-review outcome was a revision and papers which were still under review; thus, the dataset for this analysis comprises 20,706 records of which 8934 were accepted and 11,772 were rejected. The decision post-review of whether to accept a paper or not is taken by the editor but is based on the feedback received from the referees, so we assume that the decision at this stage would reflect a potential referee bias.

Table 11 displays the accept rate by review type defined as the number of accepted papers over the total number of accepted or rejected papers.

**Table 11:** Acceptance rate by review type

Outcome	DBPR		SBPR	
Accepted	242 (25%)		8692 (44%)	
	Nature	24	Nature	1116
	Nature Communications	137	Nature Communications	4077
	Sister journals	81	Sister journals	3499
Rejected	732 (75%)		11,040 (56%)	
	Nature	108	Nature	2226
	Nature Communications	294	Nature Communications	3843
	Sister journals	330	Sister journals	4971

We found that DBPR papers that are sent to review have an acceptance rate that is significantly lower than that of SBPR papers. The results of a Pearson’s chi-square test of independence show a small effect size (*χ*^2^ = 138.77, df = 1, *p* value < 0.001; Cramer’s *V* = 0.082).

We investigated the question of whether, out of the papers that go to review, manuscripts by female corresponding authors are more likely to be accepted than those with male corresponding authors under DBPR and SBPR. We excluded the records for which the assigned gender was NA and focussed on a dataset of 17,167 records, of which 2849 (17%) had a female corresponding author and 14,318 (83%) had a male corresponding author. First, we calculated the acceptance rate by gender, regardless of review type (Table 12).

**Table 12:** Outcome for papers sent to review as a function of the gender of the corresponding author, regardless of review model

Outcome	Female corresponding authors	Male corresponding authors	NA value for gender of corresponding authors
Accepted	1222 (43%)	6434 (45%)	1278 (36%)
Rejected	1627 (57%)	7884 (55%)	2261 (64%)

We decided to exclude the gender values NA and we observed a significant but very small difference in the acceptance rate by gender (Pearson’s chi-square test of independence: *χ*^2^ = 3.9364, df = 1, *p* value = 0.047; Cramer’s *V* = 0.015), leading us to conclude that manuscripts by female corresponding authors are slightly less likely to be accepted. We identify two potential causes for this, one being a difference in quality and the other being a gender bias.

To ascertain whether indeed any referee bias is present, we studied the acceptance rate by gender and review type. Table 13 shows the proportion of manuscripts that are sent for review and accepted or rejected with different peer review model and by gender of the corresponding author.

**Table 13:** Outcome of papers sent to review by gender of the corresponding author and by review model

Outcome	Female corresponding authors		Male corresponding authors		NA value for gender of corresponding authors	
	DBPR	SBPR	DBPR	SBPR	DBPR	SBPR
Accepted	35 (26%)	1187 (44%)	137 (25%)	6297 (46%)	70 (24%)	1208 (37%)
Rejected	99 (74%)	1528 (56%)	413 (75%)	7471 (54%)	220 (76%)	2041 (63%)

If we compare the proportion of accepted manuscripts under DBPR and authored by female vs. male corresponding authors (26 vs. 25%) with a test for equality of proportions with continuity correction, we find that there is a not a significant difference in female authors and male authors for DBPR-accepted papers (results of two-sample test for equality of proportions with continuity correction: *χ*^2^ = 0.03188, df = 1, *p* value = 0.8583).

If we compare male authors’ and female authors’ acceptance rates for SBPR papers (44 vs. 46%), we find that there is not a significant difference in female authors and male authors for SBPR-accepted manuscripts (results of two-sample test for equality of proportions with continuity correction test: *χ*^2^ = 3.6388, df = 1, *p* value = 0.05645). Based on these results, we cannot conclude whether the referees are biased towards gender.

In order to detect any bias towards institutional prestige, we referred to a dataset containing 20,706 records, which includes OTR papers that were either rejected or accepted, as well as transfers. Table 14 shows acceptance rate by institution group, regardless of review type.

**Table 14:** Outcome of manuscripts sent to review as a function of the institution group of the corresponding author, regardless of review model

Outcome	Institution group 1	Institution group 2	Institution group 3	Institution group 4
Accepted	996 (49%)	2108 (43%)	2078 (40%)	3752 (43%)
Rejected	1029 (51%)	2743 (57%)	3100 (60%)	4900 (57%)

There is a small but significant association between institution group and acceptance (Pearson’s chi-square test results: *χ*^2^ = 49.651, df = 3, *p* value < 0.001, Cramer’s *V* = 0.049). This result does not change significantly if we focus on the three institution groups we defined (high-, medium-, and low-prestige), thus excluding the fourth group for which no THE rank was found (Pearson’s chi-square test results: *χ*^2^ = 49.405, df = 2, *p* value < 0.001, Cramer’s *V* = 0.064), which means that authors from less prestigious institutions tend to be rejected more than authors from more prestigious institutions, regardless of review type. The difference, however, is very small.

In order to measure any quality effect, we tested the null hypothesis that the populations (institution group 1, 2, and 3) have the same proportion of accepted manuscripts for DBPR manuscripts with a test for equality of proportions (proportion of accepted manuscripts 0.37 for group 1, 0.31 for group 2, and 0.23 for group 3). The test yielded a non-significant *p* value (*χ*^2^ = 5.2848, df = 2, *p* value = 0.07119).

We tested the null hypothesis that the populations (institution groups 1, 2, and 3) have the same proportion of accepted manuscripts for SBPR manuscripts with a test for equality of proportions (proportion of accepted manuscripts 0.49 for group 1, 0.44 for group 2, and 0.41 for group 3). We found a significant result (*χ*^2^ = 37.76, df = 2, *p* value < 0.001). This means that there is a statistically significant difference between the three groups. In order to identify the pair(s) giving rise to this difference, we performed a test of equal proportion for each pair and accounted for multiple testing with Bonferroni correction. The results were significant for all pairs: group 1 vs. group 2 (*χ*^2^ = 15.961, df = 1, *p* value < 0.001); group 2 vs. group 3 (*χ*^2^ = 7.1264, df = 1, *p* value = 0.0227); and group 1 vs. group 3 (*χ*^2^ = 37.304, df = 1, *p* value < 0.001). In order to see whether the final decision outcome could be accurately predicted based on author and journal characteristics, we attempted to fit logistic regression models to the data. We aimed at modelling acceptance based on the following variables (and all their subsets): review type (SB/DB), corresponding author’s gender, the group of their institution (1, 2, 3, or 4), the category of their country (Australia, Canada, China, France, Germany, India, Japan, South Korea, the UK, the USA, and Others), and the journal tier (Nature, Nature sister journals, and Nature Communications). Similar to the uptake case, the models do not have a good fit to the data. The full model has a pseudo *R*^2^ of 0.03, and the binned plot of the model’s residuals against the expected values also shows a poor fit. The area under the receiving operating characteristic (ROC) curve is 0.40.

## Discussion

DBPR was introduced in the Nature journals in response to the author community’s wish for a bias-free peer review process. The underlying research question that drove this study is to assess whether DBPR is effective in removing or reducing implicit reviewer bias in peer review. As mentioned above and discussed below in more detail, the fact that we did not control for the quality of the manuscripts means that the conclusions on the efficacy of DBPR that can be drawn from this data are limited. Any conclusive statement about the efficacy of DBPR would have to wait until such control can be implemented or more data collected. Nevertheless, the available data allowed us to draw conclusions on the uptake of the review models, as we detail below.

Another issue that hampered our study was the lack of complete records for each manuscript in the dataset in relation to gender, country, and institution of the corresponding author. This is because the Nature journals do not collect information on author’s gender, and thus, such information can only be retrieved with name-matching algorithms with limited accuracy. As mentioned in the “Methods” section, we have used a commercial algorithm to attribute gender based on first names, and discarded records that could not be matched with accuracy greater than 80%. This can potentially skew our results if, for example, there are differences in the proportion of names that cannot be attributed between genders. Moreover, some records were not complete if authors made spelling mistakes when entering the names of their country or institution, as this would have made it impossible to match those names with normalised names for countries or for institutions using GRID. While these shortcomings of the data are beyond our control, we have made it clear in the “Results” section when and why we have excluded a subset of the dataset in each aspect of the analysis.

The results on author uptake show that DBPR is chosen more frequently by authors that submit to higher impact journals within the portfolio, by authors from certain countries, and by authors from less prestigious institutions. The proportion of authors choosing double-blind changes as a function of the institution group, with higher ranking groups having a higher proportion of single-blind manuscripts (Table 4). We did not observe gender-related differences in uptake. More specifically, the proportion of authors choosing DBPR is lower for higher ranking institution groups; in the uptake analysis by country, China and the USA stand out for their strong preference for DBPR and SBPR, respectively. In general, authors from countries with a more recent history of academic excellence are more likely to choose DBPR. These results suggest that the choice of DBPR may be linked with a higher perceived risk of discrimination, with the exception of gender discrimination. That is, authors that feel more vulnerable to implicit bias against the prestige of their institutional affiliation or their country tend to choose DBPR to prevent such bias playing a role in the editorial decision.

The available data cannot tell us if other factors, such as the quality of the work, play a role in the choice of the review model. Authors might choose SBPR when submitting their best work as they are proud of it and may opt for DBPR for work of lower quality, or, the opposite could be true, that is, authors might prefer to submit their best work as DBPR to give it a fairer chance against implicit bias. Either behaviour may apply to different demographics of authors. Because we were unable to independently measure the quality of the manuscripts, this quality-dependent selection, if present, remains undetermined in our study.

The analysis of success outcome at both the out-to-review and acceptance stages could in principle reveal the existence of any reviewer bias against authors’ characteristics. In our case, this analysis was hampered by the lack of an independent measure of quality, by potential confounders such as potential editor bias towards the review model or author characteristics, and by the lack of controlled experiments in which the same paper is reviewed under both SBPR and DBPR, or in which DBPR is compulsory, thus eliminating the effect of bias towards the review model. We considered using citations as a proxy for the quality of published papers; however, this would have limited the dataset to the small number of published articles that have had time to accrue citations, given the low acceptance rate of the journals considered, and the fact that the dataset is recent in relation to when DBPR was introduced at the Nature journals. Controlled experiments as described above were not possible due to peer review policies at the Nature journals and the fact that we could only analyse historical data.

Our results show that we cannot say that there is a significant difference between authors from prestigious institutions and authors from less prestigious institutions for DBPR-accepted manuscripts. Moreover, DBPR manuscripts are less likely to be successful than SBPR manuscripts at both the decision stages considered (Tables 5 and 10), but because of the above limitations, our analysis could not disentangle the effects of these factors: bias (from editors and reviewers) towards various author characteristics, bias (from editors and reviewers) towards the review model, and quality of the manuscripts. The lack of a significant association between gender and OTR rate regardless of peer review model (Table 7) might suggest that there is no editor bias towards gender; however, this is based on the assumption that there is no gender-dependent quality factor. For other authors’ characteristics, such as institutional prestige, a quality factor is more likely than for gender: it is not unthinkable to assume that on average manuscripts from more prestigious institutions, which tend to have more resources, are of a higher quality than those from institutions with lower prestige and fewer means. Thus, we cannot draw conclusions on any editor bias.

In the out-to-review analysis, we observed a significant difference between the OTR rate of papers by male and female corresponding authors of DBPR papers. This might be the result of editor bias towards the review model, of the fact that female authors select their best papers to be DBPR to increase their chances of being accepted, or both. Across the three institution groups, SBPR papers are more likely to be sent to review. This may be due to editor bias towards the review model, to a quality effect (authors within each institution group choose to submit their best studies under SBPR), or both.

In the post-review analysis, we found that DBPR papers that are sent to review have an acceptance rate that is significantly lower than that of SBPR papers. This might be due to referee bias against review model, or to a lower quality of DBPR papers, or both. There is a tiny but significant association between institution group and acceptance, which means that authors from less prestigious institutions tend to be rejected more than authors from more prestigious institutions, regardless of review type. This can be due to quality or referee bias. When comparing acceptance rates by gender and regardless of review model, we observed that female authors are significantly less likely to be accepted than their male counterparts. However, we were unable to distinguish the effects of gender bias (from reviewers) and manuscript quality in this observation because an analysis of acceptance rate by gender and review type did not yield statistically significant results.

The post-review outcome of papers as a function of the institution group and review model (Table 15) showed that manuscripts from less prestigious institutions are accepted at a lower rate than those from more prestigious ones, even under DBPR; however, due to the small numbers of papers at this stage, the results are not statistically significant. This may occur as a consequence of positive referee bias towards institution groups or to quality factors.

**Table 15:** Outcome of manuscripts sent to review as a function of the institution group of the corresponding author and review model

Outcome	Institution group 1		Institution group 2		Institution group 3	
	DBPR	SBPR	DBPR	SBPR	DBPR	SBPR
Accepted	11 (37%)	985 (49%)	61 (30%)	2047 (44%)	75 (23%)	2003 (41%)
Rejected	19 (63%)	1010 (51%)	139 (70%)	2604 (56%)	251 (77%)	2849 (59%)

We should note that the significance of the results on outcome is limited by the size of the dataset for accepted papers, due to the high selectivity of these journals and to the low uptake of DBPR. We calculated that, at this rate, it would take us several decades to collect sufficient data that would result in statistically significant results, so another strategy is required, e.g. making DBPR compulsory to accelerate data collection and remove potential bias against the review model. Also, because of the retrospective nature of this study, we could not conduct controlled experiments. In future works, we will consider studying the post-decision outcome also in relation to the gender of reviewers and defining a quality metric for manuscripts in order to isolate the effect of bias.

The multivariate regression analyses we performed led to uninformative models that did not fit the data well when the response was author uptake, out-to-review decision, or acceptance decision, and the predictors were review type, author gender, author institution, author country, and journal tier. Since the models showed a bad fit to the data according to accepted diagnostics criteria, further interpretation of the models is not warranted. One possible explanation for the lack of fit is that more or other predictors would be needed in order to fully explain the response, for example, a measure of quality, as we have already indicated. Another possibility is that the predictors are correlated, thus preventing a good fit. In our case, the option that the outcome is subject to a complex combination of soft constraints or incentives is possible, which supports our simpler approach of evaluating the variables with the bivariate approach we have reported on.

## Conclusions

This study is the first one that analyses and compares the uptake and outcome of manuscripts submitted to scientific journals covering a wide range of disciplines depending on the review model chosen by the author (double-blind vs. single-blind peer review). We have analysed a large dataset of submissions to 25 Nature journals over a period of 2 years by review model and in dependence of characteristics of the corresponding author. Our aim was to understand the demographics of author uptake and infer the presence of any potential implicit bias towards gender, country, or institutional prestige in relation to the corresponding author.

This study provides insight on author’s behaviour when submitting to high-impact journals. We observed that DBPR is chosen more often by authors submitting to higher impact journals within the Nature portfolio, by authors from specific countries (India and China in particular, among countries with the highest submission rates), and by authors from less prestigious institutions. We did not observe any difference by author gender. We found that manuscripts submitted under DBPR are less likely to be sent to review and accepted than those submitted under SBPR. We also found that manuscripts from female authors or authors from less prestigious institutions are accepted with a lower rate than those from male authors or more prestigious institutions, respectively.

Because of the small size of the data set for accepted papers and of the lack of an independent measure for the quality of the manuscripts, we could not draw firm conclusions on the existence of implicit bias and on the effectiveness of DBPR in reducing or removing it.

## Abbreviations

DBPR: Double-blind peer review
GRID: Global Research Identifier Database
OTR: Out to review
SBPR: Single-blind peer review
THE: Times Higher Education
